# Type IV pityriasis rubra pilaris treated with ixekizumab

**DOI:** 10.1016/j.abd.2025.501283

**Published:** 2026-01-14

**Authors:** Ana Ferreirinha, Alexandre João, Margarida Brito Caldeira

**Affiliations:** Dermatology and Venereology Department, Hospital de Santo António dos Capuchos, Unidade Local de Saúde São José, Lisbon, Portugal

Dear Editor,

Juvenile Pityriasis Rubra Pilaris (PRP) is a rare inflammatory dermatosis of unknown etiology, presenting diagnostic and therapeutic challenges.^1^ Despite a recognized peak incidence in early childhood, reports on the clinical management of pediatric PRP, particularly with newer biologic therapies, remain scarce. Recent studies have implicated immunologic pathways, particularly Interleukins (IL) 17 and 23, in the pathogenesis of PRP, suggesting potential targets for therapeutic intervention.^1^

An 8-year-old girl presented with a two-week history of progressive lesions, initially localized to the soles and subsequently extending to the palms, face, scalp, knees, elbows, and occipital and anogenital region. Symptoms included pruritus without diurnal variation. She had neither history of febrile episodes, recent medication use or vaccination, nor family history of dermatological disorders. Clinical examination revealed diffuse, symmetrical, orange, waxy palmoplantar keratoderma with fissures, accompanied by erythematous, scaly plaques with follicular accentuation on the elbows, knees, anogenital, face, scalp, and auricular areas (Fig. 1A‒D). Histopathological findings confirmed PRP, showing characteristic focal and confluent hypergranulosis with alternating orthokeratosis and parakeratosis. Based on the clinical presentation and the histopathological findings, the patient was diagnosed with juvenile PRP type IV (circumscribed juvenile form). Initial treatment with high-potency topical corticosteroids yielded no improvement. Methotrexate 15 mg/week alongside a short course of prednisolone (10 mg/day) also proved ineffective. Due to the disease's severity and impact on quality of life, off-label ixekizumab (with an 80 mg loading dose) was introduced, in addition to methotrexate. Within four weeks of initiating therapy, the patient showed significant improvement. By the five-month follow-up, only post-inflammatory hypopigmentation remained on the knees and elbows, with complete resolution of lesions in the anogenital, face and scalp regions and a marked reduction in palmoplantar keratoderma infiltration (Fig. 2A‒D). She successfully discontinued topical corticosteroids, began tapering methotrexate, and continues on ixekizumab (40 mg every four weeks) without any reported adverse effects, leading to a substantial improvement in both her quality of life and that of her family.

**Figure 1:**
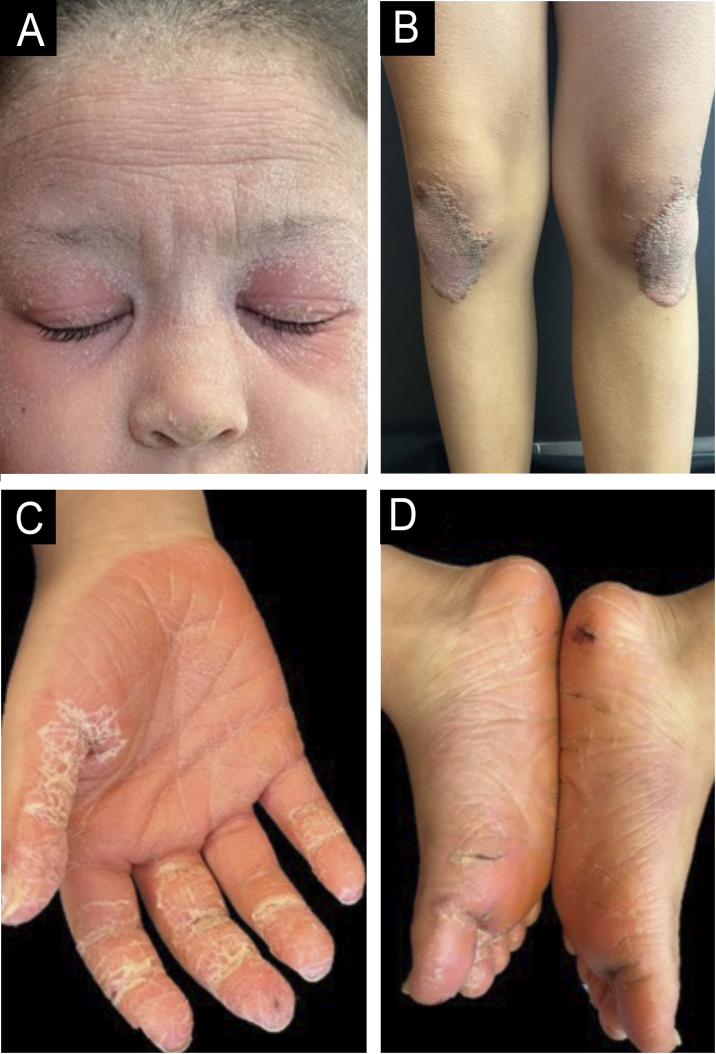
(A‒B) Erythematous, scaly plaques with follicular accentuation on the face and knees. (C‒D) Diffuse, symmetrical, orange, waxy palmoplantar keratoderma with fissures.

**Figure 2:**
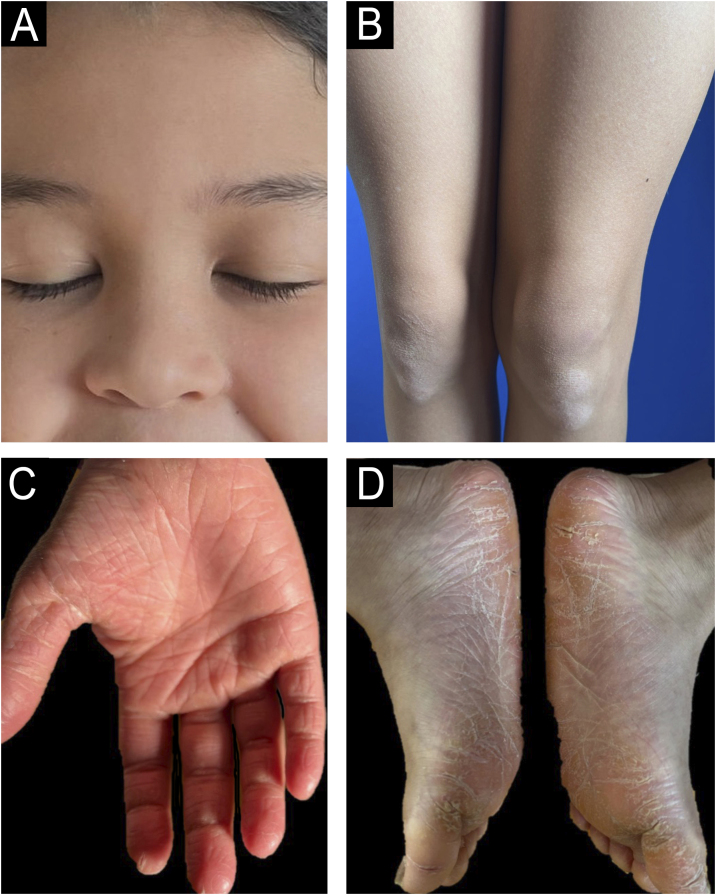
(A‒B) By the five-month follow-up, only post-inflammatory hypopigmentation remained on the knees, with complete resolution in the face and scalp. (C‒D) Marked reduction in palmoplantar keratoderma.

Given the heterogeneity of PRP, there is no universal treatment approach.^1^ In our case, systemic retinoids were avoided due to the risk of premature epiphyseal closure. Instead, methotrexate was initiated as a second-line therapy; however, no response was observed after two months. Biologic therapies approved for psoriasis have been increasingly used in treatment-refractory PRP cases.^1^

Among anti-TNF-alpha agents, etanercept and adalimumab have been used to treat pediatric PRP, along with ustekinumab.^1^ Responses have been variable; for instance, although many patients treated with ustekinumab showed impressive improvement, not all experienced sustained or durable responses.^1^ To our knowledge, no cases of pediatric PRP treated with anti-IL-23 agents have been reported to date.

Regarding anti-IL-17 therapies, their mechanism of action in PRP likely involves the inhibition of IL-17-mediated keratinocyte proliferation and inflammatory pathways.^1^, ^2^ Ixekizumab, an anti-IL-17A antibody, is FDA-approved for the treatment of moderate to severe plaque psoriasis in children aged six years and older. While it has demonstrated efficacy in adult PRP, its safety and effectiveness in pediatric PRP remain to be fully established.^1^ Similarly, secukinumab, another anti-IL-17A antibody, has shown efficacy in both adult PRP and in a reported case of type V PRP (atypical juvenile form).^1^ Our case demonstrated remarkable subjective and objective improvement with ixekizumab, further supporting the only other reported case in the literature.^3^ Ixekizumab was preferred over secukinumab because its single loading dose is more convenient than secukinumab’s four weekly injections, making it a more practical choice for children. Regarding drug discontinuation, data on relapse rates in PRP remain limited. Based on the natural history of juvenile type IV PRP, the prognosis is uncertain; however, a proportion of cases resolves within 1–3 years of onset, with some achieving complete remission by late adolescence.^2^ Considering the clinical context, ixekizumab therapy will be maintained with a planned gradual tapering regimen, individualized according to the patient’s tolerability and disease control, with close surveillance for therapeutic response, adverse events, and risk of recurrence.

This case highlights the successful off-label use of ixekizumab in pediatric PRP, demonstrating its potential as a safe and effective treatment option. It offers a promising alternative to conventional therapies such as methotrexate and acitretin, which are frequently associated with significant toxicity and limited tolerability in children.

## ORCID ID

Ana Ferreirinha: 0009-0008-1336-3923

Alexandre João: 0000-0002-2517-9604

Margarida Brito Caldeira: 0000-0002-2538-4739

## Research data availability

Does not apply.

## Financial support

None declared.

## Authors' contributions

Ana Ferreirinha: Conception and design of the study; Data collection, analysis, and interpretation; Drafting or critical revision of the manuscript for important intellectual content; Acquisition, analysis, and interpretation of clinical data; Active participation in the guidance of the research; Intellectual involvement in the diagnostic and/or therapeutic management of the case; Critical review of the literature; Final approval of the submitted version of the manuscript.

Alexandre João: Conception and design of the study; Data collection, analysis, and interpretation; Drafting or critical revision of the manuscript for important intellectual content; acquisition, analysis, and interpretation of clinical data; Active participation in the guidance of the research; Intellectual involvement in the diagnostic and/or therapeutic management of the case; Critical review of the literature; Final approval of the submitted version of the manuscript.

Margarida Brito Caldeira: Conception and design of the study; Data collection, analysis, and interpretation; Drafting or critical revision of the manuscript for important intellectual content; Acquisition, analysis, and interpretation of clinical data; Active participation in the guidance of the research; Intellectual involvement in the diagnostic and/or therapeutic management of the case; Critical review of the literature; Final approval of the submitted version of the manuscript.

## Conflicts of interest

None declared.
